# Motor Adaptation to Cognitive Challenges and Destabilizing Treadmill Walking in Healthy Older Adults

**DOI:** 10.1093/geroni/igaa057.1687

**Published:** 2020-12-16

**Authors:** Pei-Chun Kao, Michaela Pierro

**Affiliations:** 1 University of Massachusetts Lowell, Lowell, Massachusetts, United States; 2 Delsys Inc, Natick, Massachusetts, United States

## Abstract

To develop effective fall prevention intervention, it is necessary to understand how older adults respond to challenges that demand cognitive-motor dual-tasking capability, an important capability in the daily lives. The purpose of this study is to investigate how older adults adjust their motor responses when encountering cognitive and walking perturbations simultaneously. We recorded kinematic data as subjects walked on a treadmill with or without 1) continuous random-amplitude treadmill platform sways (Perturbed vs. No-perturbed walking); and 2) each of the four cognitive tasks: Paced Auditory Serial Addition test (PASAT), clock test, visual color-word incongruent test (V-stroop), and auditory pitch-word incongruent test (A-stroop). We computed dynamic margins of stability (MOS), gait variability, and short-term local divergence exponent (LDE) of the trunk motion (local stability). Data of ten older subjects (age: 72.2±4.9) show that cognitive performance did not differ between standing, Perturbed or No-perturbed walking. Subjects demonstrated significantly greater local instability and variability in step measures, joint angle and MOS during Perturbed than No-perturbed walking (p<0.001). During dual-task conditions, subjects walked with significantly larger medio-lateral MOS (MOSML) compared to walking only, especially during early phase of the trial. During Perturbed walking, subjects had significantly larger MOSML during PASAT and Vstroop than walking only. Our data showed that subjects tried to increase their dynamic MOS during Perturbed walking or a cognitive task more difficult or taxing visual attention. However, the adjustments do not sustain throughout the trial. These findings suggest older adults tend to prioritize cognitive over walking tasks even when encountering walking perturbations.

